# Body Inversion Effects With Photographic Images of Body Postures: Is It About Faces?

**DOI:** 10.3389/fpsyg.2019.02686

**Published:** 2019-11-29

**Authors:** Emma L. Axelsson, Rachel A. Robbins, Helen F. Copeland, Hester W. Covell

**Affiliations:** ^1^School of Psychology, The University of Newcastle, Callaghan, NSW, Australia; ^2^Research School of Psychology, The Australian National University, Canberra, ACT, Australia

**Keywords:** body representations, inversion effects, eye tracking, faces, headless bodies

## Abstract

As with faces, participants are better at discriminating upright bodies than inverted bodies. This inversion effect is reliable for whole figures, namely, bodies with heads, but it is less reliable for headless bodies. This suggests that removal of the head disrupts typical processing of human figures, and raises questions about the role of faces in efficient body discrimination. In most studies, faces are occluded, but the aim here was to exclude faces in a more ecologically valid way by presenting photographic images of human figures from behind (about-facing), as well as measuring gaze to different parts of the figures. Participants determined whether pairs of sequentially presented body postures were the same or different for whole and headless figures. Presenting about-facing figures (heads seen from behind) and forward-facing figures with faces enabled a comparison of the effect of the presence or absence of faces. Replicating previous findings, there were inversion effects for forward-facing whole figures, but less reliable effects for headless images. There were also inversion effects for about-facing whole figures, but not about-facing headless figures. Accuracy was higher in the forward- compared to the about-facing conditions, but proportional dwell time was greater to bodies in about-facing images. Likewise, despite better discrimination of forward-facing upright compared to inverted whole figures, participants focused more on the heads and less on the bodies in upright compared to inverted images. However, there was no clear relationship between performance and dwell time proportions to heads. Body inversion effects (BIEs) were found with about-facing whole figures and headless forward-facing figures, despite the absence of faces. With inverted whole figures, there was a significant relationship between performance and greater looking at bodies, and less at heads suggesting that in more difficult conditions a focus on bodies is associated with better discrimination. Overall, the findings suggest that the visual system has greater sensitivity to bodies in their most experienced form, which is typically upright and with a head. Otherwise, the more a face is implied by the context, as in whole figures or forward- rather than about-facing headless bodies, the better the performance as holistic/configural processing is likely stronger.

## Introduction

Inverted faces are more difficult to discriminate than upright faces, and this inversion effect is larger than that seen with other objects such as dogs or houses (e.g., Diamond and Carey, 1986; Farah et al., 1995; Rossion, 2008). Explanations for the effect vary. One argument is that faces are a unique category subject to specialized processing, perhaps because we are highly familiar with them, and they share the same first-order configuration (eyes above nose above mouth), which means that telling them apart is based not just on the presence of certain features, but also holistic or configural processing (e.g., Robbins and McKone, 2007). Another category associated with equally large inversion effects is human bodies (e.g., Robbins and Coltheart, 2012b). Like faces, exposure to bodies is highly frequent, and they also share a first-order configuration (head on body, typically two arms on the sides, and two legs below). However, bodies are attached to faces, so one question is whether or how much the body inversion effect (BIE) is influenced by actual or induced face information when discriminating bodies.

Reed et al. (2003, 2006) were the first to report a BIE. Participants discriminated sequentially presented pairs of 3D software-created images of body postures (i.e., not natural bodies). For half of the pairs, the arm, leg, and head positions differed slightly and participants judged whether the pairs were the same or different. Participants were slower and less accurate for inverted compared to upright postures and the inversion effect was similar in magnitude to that seen on a facial identity task. Multiple studies have replicated Reed et al.’s (2003, 2006) findings with similar stimuli (Brandman and Yovel, 2010; Yovel et al., 2010, Experiment 1). BIEs have also been found in body identity discrimination tasks using photographic images, such that people were more accurate (Robbins and Coltheart, 2012b) and more efficient (inverse efficiency = RT/accuracy; Minnebusch et al., 2009) when discriminating upright compared to inverted images of people.

Perhaps surprisingly, early studies found no BIE for bodies WITHOUT heads (headless bodies). In an identity discrimination task, Minnebusch et al. (2009) found no inversion effect with upright and inverted headless bodies in accuracy or reaction time (RT), and a reversed BIE in efficiency such that participants were more efficient at discriminating *inverted* headless bodies than upright. Similarly, Yovel et al. (2010, Experiment 2) failed to find a BIE for posture discrimination with headless bodies for either accuracy (*d*’) or RT (see also behavioral data in Brandman and Yovel, 2010, 2012). They further tested whether the BIE would be reduced when *any* body part is removed, not just the head, as we typically see human figures in their complete form. Figures presented without arms or missing a leg still led to BIEs. When the heads on the pairs of figures in the sequential matching tasks were in identical as opposed to variable positions, the BIE was reduced in magnitude suggesting that head positions contribute to the discriminatory process. Therefore, the failure to find a BIE was not due to the figures appearing in an incomplete form, but rather due to the absence of heads. Yovel et al. (2010) argued that the BIE was based on the presence of a head, such that it could be explained by the body activating face sensitive areas in the brain associated with a face inversion effect (FIE).

Brandman and Yovel (2012) further investigated the importance of a face to the BIE by presenting whole figures of people from behind (i.e., about-facing) again in a posture task. For these about-facing whole figures, a BIE was found, but it was significantly smaller in magnitude to that seen with forward-facing (faceless) whole figures. This study replicated a failure to find a BIE for forward-facing headless bodies. They also found FIEs for faceless heads, and faceless heads presented with upper torsos. Interestingly, following brief presentations of upright figures (27 milliseconds), participants were more likely to rate themselves as having *seen* a face in the faceless, forward-facing whole figures than in the headless figures, and they were least likely to rate themselves as having perceived a face in the about-facing whole figures. Brandman and Yovel (2010) argued that the more likely a face is induced by the contextual information in the stimuli, the more likely an inversion effect is found. However, it is somewhat surprising that a BIE is found with about-facing whole figures raising questions about the role of the implied existence of facial features in contributing to a BIE. Note, about-facing headless bodies were not presented presumably due to a lack of a BIE with forward-facing headless bodies. Including a headless about-facing condition would further test if induced facial information is key for a BIE.

Finally, in an fMRI paradigm, Brandman and Yovel (2010) measured differences in activation to pairs of different and same body postures in face-selective areas [fusiform face area (FFA) and occipital face area (OFA)] and body selective areas of the brain [extrastriate body area (EBA) and fusiform body area (FBA)]. A greater response to the different compared to the same posture pairs is suggestive of greater sensitivity. Brandman and Yovel (2010) found that face selective areas were only sensitive to (faceless) whole figures, but not headless bodies; whereas body-selective areas were sensitive to the presentations of whole figures and headless bodies in both upright and inverted orientations. In particular, the face-selective areas only demonstrated sensitivity to the upright, but not the inverted whole figures. Brandman and Yovel (2010) argued that this pattern of brain activation could explain why information from heads is critical to the BIE. Note, however, as about-facing whole figures were not presented in this study, it is uncertain as to whether face-selective areas are also sensitive to upright, about-facing whole figures.

However, the story for headless bodies is more complicated than these early studies imply. In an identity discrimination task, Robbins and Coltheart (2012b) found a significant BIE for headless bodies in accuracy in two experiments, although the inversion effect for headless bodies was smaller than for whole figures for unfamiliar bodies. More recently, Arizpe et al. (2017), using Yovel et al.’s (2010) stimuli, found a BIE with headless stimuli (with *d*’), but performance was weaker than that seen with whole figures and the inversion effect for whole and headless bodies in RT was similar, and both significant.

How can Yovel et al.’s (2010) and Brandman and Yovel’s (2010, 2012) findings be reconciled with Arizpe et al. (2017) who found a headless body posture BIE, and Robbins and Coltheart (2012b) who found a headless body identity BIE, or even Minnebusch et al.’s (2009) reversed headless BIE? Yovel et al.’s (2010) failure to find a headless BIE could be due to reduced statistical power to find a real but small effect, as they had only *n* = 12 per condition. Brandman and Yovel (2012) had slightly more participants with *n* = 14 per condition. Arizpe et al. (2017) had a slightly larger sample of *n* = 16, but still found weaker performance than that seen with whole figures. Minnebusch et al. (2009) found no or a reversed BIE for headless bodies, depending on the dependent variable, with *n* = 17. Robbins and Coltheart (2012b) had *n* = 24 in their familiarized bodies experiment (Experiment 1) and *n* = 40 in their unfamiliar bodies experiment (Experiment 2). Susilo et al. (2013) found BIEs with whole and headless bodies, also with Yovel et al.’s (2010) stimuli, in a small sample of participants (3 out of 4) with acquired prosopagnosia (condition involving a difficulty in recognizing faces). In a follow-up study, Quigan et al. (in preparation) found a BIE for whole figure and headless bodies with an even larger sample size amongst participants with (*n* = 70+) and without developmental prosopagnosia (*n* = 70+). Effect sizes in Yovel et al. (2010; Cohen’s *d*) ranged from 1.7 for armless to 4.5 for whole figures with varied heads, but the key effect size for headless bodies is not provided. The other studies cited here did not provide effect sizes. It does seem, however, that when the sample sizes were larger, a headless BIE is found (Robbins and Coltheart, 2012b; Arizpe et al., 2017; Quigan et al., in preparation). The current study had *n* = 28 in each condition to ensure that any null results for headless bodies would be more reliable.

Another question about Yovel et al.’s (2010) and Brandman and Yovel’s (2010, 2012) studies is that the stimuli were not real, but instead 3D-software created bodies. Observers might be more willing to suspend their disbelief at the sight of a headless or an inverted body. This cannot be the only reason that Yovel et al. (2010) did not find a BIE for headless bodies, as Arizpe et al. (2017) found a BIE with the same stimuli, and Minnebusch et al. (2009) did not find a BIE with photographs of real people. However, one issue with Minnebusch et al.’s (2009) stimuli is that the test pairs were not matched in clothes and hair and responses could have been based more on these differences than on identity information. Further, in Brandman and Yovel’s (2012) study, examining whether an implied face leads to a BIE, they presented whole figures with faces occluded, headless bodies (forward-facing) and whole figures from behind (about-facing). This did not allow a direct comparison of whole figures WITH faces to whole figures without a face or forward- and about-facing headless bodies. By presenting people from behind, a face is less expected, as was found by Brandman and Yovel (2012) with about-facing whole figures. However, they still found a BIE with about-facing whole figures. Perhaps the presence of a head, albeit from behind, still induces an implied presence of a face, which in turn contributes to a BIE. Bodies are also seen with heads suggesting that whole figure inversion effects could also be partly explained by experience. The current study thus used photographs of real people seen from the front and behind (forward- and about-facing), in both whole figures and headless versions to allow direct comparisons of responses to images with and without faces that were also with and without heads.

The current study used a posture discrimination task using sequential matching, and given the previous findings (Reed et al., 2006; Brandman and Yovel, 2010, 2012; Yovel et al., 2010; Arizpe et al., 2017), a BIE was expected with forward-facing whole figures. Including about-facing whole figures might more directly address the role of faceless figures in a more ecologically valid way, one that does not involve occluding facial features. Aside from Brandman and Yovel (2012), there are no other known studies involving about-facing images; and no known studies presenting about-facing headless stimuli. If Yovel et al. (2010) are correct, and the BIE for whole figures is based on an induced face, then we expected a BIE for the about-facing whole figures given the presence of a head. Given the inconsistent findings in previous studies (e.g., Yovel et al., 2010; Arizpe et al., 2017) it was uncertain as to whether BIEs would be seen with headless images and if the effects would vary for forward- and about-facing images. Given that participants in Brandman and Yovel (2010) were less likely to rate themselves as having seen a face in both the (forward-facing) headless and about-facing whole figures, it would be highly unlikely that a face is perceived in headless about-facing images. If the BIE is based on the induced presence of a face a smaller or no BIE was expected for the headless stimuli, in particular the about-facing headless stimuli. However, a BIE for forward-facing headless stimuli was also considered possible given that Brandman and Yovel (2010) found that participants still perceived faces in these images (albeit weakly) and others have found a BIE with forward-facing headless stimuli (e.g., Arizpe et al., 2017).

We also measured where people looked for whole versus headless, front- versus about-facing figures, and upright versus inverted images. Arizpe et al. (2017) found that participants tended to look longer at the upper regions of the upright whole figures such that they looked longer at the upper torso and heads, and for inverted figures they looked longer at the lower torso. When instructed to look at the heads or the upper torso of figures in both orientations, performance was better than when looking at the lower portions of the figures. All of Arizpe et al.’s (2017) images were forward-facing with faces occluded. We compared how much participants focused on heads in forward-facing images (with faces) and about-facing images (without faces), and how much people focus on bodies given that it was a body discrimination task in images with matching heads. Looking times to feet were analyzed as the feet in the inverted images appear in the upper region of the screen. The feet are part of the body posture and one question was whether a focus on this upper region of inverted images contributes to performance in the inverted conditions.

## Materials and Methods

### Participants

Participants took part in either the about-facing or the forward-facing condition. A power analysis based on the effect size η*_*p*_*^2^ = 0.33 found by Yovel et al. (2010) suggested a suitable sample size of 28 (α = 0.05, β = 0.95) for each condition (about- and forward-facing). The participants were recruited via the Australian National University (ANU) research participation sign-up webpage (SONA) and by word-of-mouth. In the about-facing experiment (*n* = 28), the mean age was 22.34 years (*SD* = 3.96 years, range = 18 to 34 years; 22 female, 6 male, 0 other), and 19 were Caucasian, 8 Asian, and 1 African. A further three people participated, but their data were excluded due to difficulties with eye tracking (*n* = 2) and sleepiness (*n* = 1). In the forward-facing experiment (*n* = 28), the participants had a mean age of 20.33 years (*SD* = 2.34 years, range = 18 to 30 years; 16 female, 12 male, 0 other), and 18 were Caucasian, and 10 Asian. A further five participated, but their data were excluded due to technical problems (*n* = 1) and difficulties with concentration and engagement with the task (*n* = 4). All participants had reported normal or corrected-to-normal vision and received course credit. The experiments were conducted in accordance with ethical standards and were approved by the ANU’s Human Research Ethics Committee (Protocol number 2015/183).

### Apparatus

An EyeLink 1000 (SR Research) eye tracker recorded participants’ eye movements by recording the infrared reflections from the cornea and pupil with a sampling frequency of 1000 Hz, and average spatial accuracy of 0.15°. Using the Desktop Mount set-up, participants’ heads were stabilized with a chin-rest positioned 90 cm from the display and 70 cm from the camera. The eye tracking camera was positioned directly in front of and beneath a 24-inch Dell Monitor with a resolution of 1920 × 1080 pixels, and 60 Hz refresh rate.

### Stimuli

**FIGURE 1:**
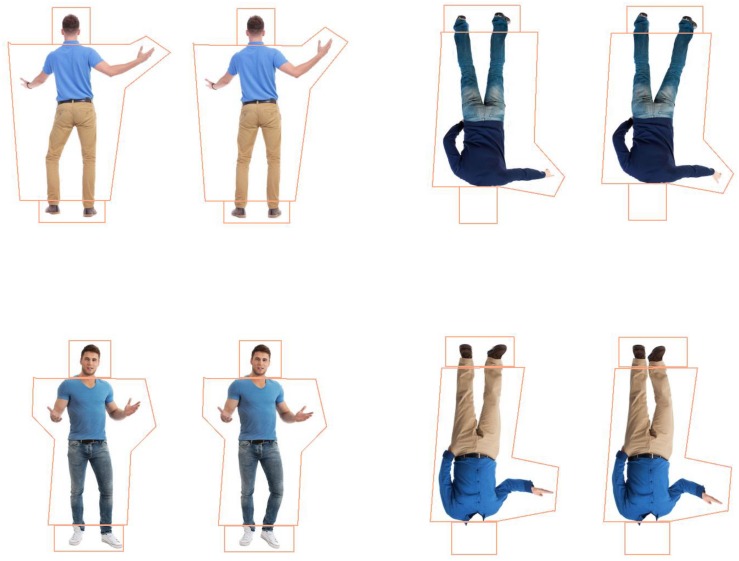
About-facing whole figure upright (WFU) and headless inverted (HLI) stimuli (top) and forward-facing WFU and HLI stimuli (bottom) with interest areas (IAs) surrounding the heads, bodies, and feet.

High quality photographs of 16 pairs of about-facing and 16 pairs of forward-facing adult, male figures were sourced from Shutterstock^1^ (see Figure 1). This necessarily meant that the identities of the about- and forward-facing bodies were different and the poses were slightly different. Only male figures in similar clothing (jeans/trousers and t-shirt, long-sleeved shirt or jumper/sweater) were used to attempt to reduce attention to clothing. Importantly, each pair had the same clothing and differed only in posture (see Figure 1). All had short hair and similar body shapes with only mild variations in weight. Four versions of each pair were created using Adobe Photoshop (CS6), one for each condition: whole figure upright (WFU), whole figure inverted (WFI), headless upright (HLU), and headless inverted (HLI) resulting in 64 stimuli pairs in each facing direction (about- or forward-facing, see Figure 1). Each pair of whole figure stimuli had an identical head; only the body postures differed. Headless stimuli were created by removing the head of the whole figure stimuli from the top of the upper garment of clothing (see Figure 1). Images were rotated 180° to create inverted stimuli. In the about-facing condition, all images were people photographed from behind. The average size of the whole figures was 8.44 × 12.84°, and the headless figures, 8.44°× 11.36° at a 90 cm distance. In the forward-facing condition, all images were photographs of people from a frontal view with faces visible in the whole figure conditions; and the average size of the whole figures was 7.33°× 13.11°, and the headless figures, 7.33°× 11.23° and the (see Figure 1). The postures were altered in 2D space by rotating or shifting the limbs of the figures up or down using Adobe Photoshop (CS6). They were divided into three categories based on the type of change made to create different postures. In each facing direction (about- and forward-facing), six pairs had a leg and an arm rotated, five pairs had only a leg rotated, and five pairs had only an arm rotated. All poses were deemed biologically possible by authors ELA and HFC. The degree of limb rotation performed in Photoshop between the initial image and the test image of each pair was 10°–15° in the “leg and arm” category, and 20°–30° in both the “arm-only” and “leg-only” categories. A smaller degree of limb rotation was used in the “leg and arm” category as the difference in postures were in two limbs, as opposed to one limb in the other categories. Head positions were identical between the pairs.

### Procedure and Design

Participants’ fixations were calibrated and validated using the standard EyeLink 1000 nine-point display. The experiment commenced once validation values of the calibration points were less than 1° visual angle. The experimenter provided instructions (orally) and written instructions also appeared on the display monitor prior to eight practice trials. The practice stimuli did not appear in the main experiment. Each trial began with a “drift correct” calibration point presented in the center of the display to ensure the participants’ fixations remained calibrated throughout. Using a sequential matching method, participants saw an initial posture from a given pair for 250 ms, followed by a 1000 ms inter-stimulus interval (ISI, a blank white screen). The test image appeared and remained on the screen until participants indicated using a keyboard if the body posture was the same or different as the initial image. Velcro was attached to two keys as tactile reminders of which keys corresponded with the “same” (smooth, “z” key) or “different” options (rough, forward slash key). Participants had 5000 ms to respond before the trial terminated and a “no-response” was recorded. Any “no-response” trials were excluded. In the forward- and about-facing conditions, respectively, participants saw all 64 pairs of stimuli, and each participant saw each image in a pair serve as the initial image or test image an equal number of times. Participants saw all four body type conditions (WFU, WFI, HLU, and HLI). There were four versions of the task which counterbalanced the order of presentation of the conditions. Trials were termed “same” or “different” depending on whether a change in body posture was present and each image appeared in an equal number of same or different trials. The same/different status of trials was randomized, as was which of the two images within each pair was presented first. A given pair appeared only once every eight trials to ensure the presentation of individual pairs was spread out. Participants took approximately 15 min to complete all trials.

## Results

Data was extracted using Data Viewer software version 1.10.1630 (SR Research). The data were analyzed using JASP 0.10.0.0. Both *d*’ and inverse efficiency scores were the main dependent variables. Signal detection sensitivity (SDT, *d*’, see Stanislaw and Todorov, 1999 for a review) was used to analyze accuracy in discriminating body postures as it incorporates correct and incorrect responses. More specifically, signal sensitivity (*d*’) indicates the difference between each participant’s standardized mean hit rate (proportion of correct responses in trials with “same” postures) and standardized mean false-alarm rate [proportion of incorrect responses in trials with “different” postures, *d*’ = *z*(hit rate) – *z*(false alarm rate)]. Larger *d*’ scores indicate a stronger recognition of change in body signal, and consequently, better performance. Response bias (criterion *c*) is a measure of participants’ tendency to be conservative and report no change (i.e., same) in body posture across both same and different trials. Efficiency was also analyzed to account for speed/accuracy trade-offs and was calculated by dividing the mean RTs in correct trials by the proportion of correct responses for each participant in each condition (Minnebusch et al., 2009; Bruyer and Brysbaert, 2011). To directly test for inversion effects, planned paired *t*-tests were also performed comparing performance between the upright and inverted images for each body type (for the whole figure and headless figures in the about- and forward-facing conditions).

### *d* Prime

A 2 × 2 × 2 mixed model ANOVA comparing *d*’ across the two facing directions (about-facing, forward-facing), the two body types (whole figure, headless), and the two orientations (upright, inverted) revealed that there was a main effect of facing direction, *F*(1,54) = 19.76, *p* < 0.001, η*_*p*_*^2^ = 0.27. Participants were overall more accurate in the forward-facing (*M* = 1.58, *SD* = 0.94) than in the about-facing condition (*M* = 0.96, *SD* = 0.68). The main effect of body type was non-significant, *F*(1,54) = 0.09, *p* = 0.770, η*_*p*_*^2^ = 0.01, but there was a significant main effect of orientation, *F*(1,54) = 10.99, *p* = 0.002, η*_*p*_*^2^ = 0.17. *d*’ scores were overall higher in the upright (*M* = 1.47, *SD* = 0.82) compared to the inverted conditions (*M* = 1.08, *SD* = 0.80). The interaction between facing direction and body type was non-significant, *F*(1,54) = 0.07, *p* = 0.937, η*_*p*_*^2^ < 0.01, but the interaction between facing direction and orientation, *F*(1,54) = 4.35, *p* = 0.042, η*_*p*_*^2^ = 0.07, and the interaction between body type and orientation were significant, *F*(1,54) = 4.85, *p* = 0.032, η*_*p*_*^2^ = 0.08. The facing direction by body type by orientation interaction was non-significant, *F*(1,54) = 0.73, *p* = 0.398, η*_*p*_*^2^ = 0.01. These interactions are explained in the following *a priori t*-tests.

In the about-facing condition, participants were more accurate (*DV* = *d*’) at detecting changes in body posture in the WFU condition (*M* = 1.15, *SD* = 0.71) compared to the WFI condition (*M* = 0.74, *SD* = 0.69), *t*(27) = −2.42, *p* = 0.023, *d* = −0.46. There was no significant difference between the HLU (*M* = 0.92, *SD* = 0.59) and HLI conditions (*M* = 1.04, *SD* = 0.73), *t*(27) = 0.78, *p* = 0.440, *d* = 0.15. Therefore, for the about-facing images, there was a BIE in the whole figure, but not the headless conditions. The effect sizes also reflect this pattern with a small-to-medium effect size for the whole figure condition and a small effect size for headless. Difference scores were then calculated between the upright and inverted conditions for the whole figure and headless conditions. A comparison of the difference scores revealed that the magnitude of the difference between the upright and inverted images (i.e., the BIE) was significantly larger in the whole figure (*M* = 0.41, *SD* = 0.89) than in the headless conditions (*M* = 0.11, *SD* = 0.82), *t*(27) = 3.86, *p* < 0.001, Bonferroni-corrected^2^ (α × 2) < 0.001, *d* = 0.74 (seeFigure 2).

**FIGURE 2:**
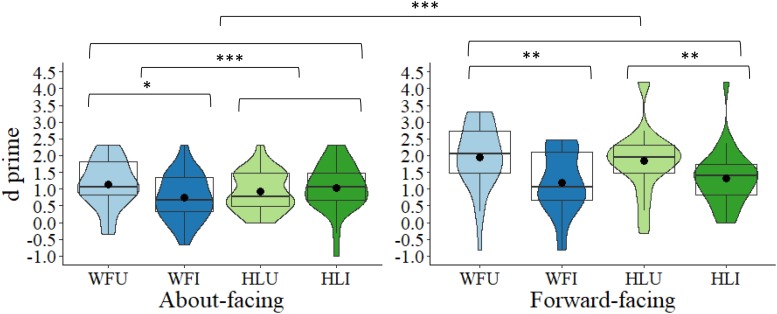
Box and violin plots of *d*’ scores for the four conditions: whole figure upright (WFU), whole figure inverted (WFI), headless upright (HLU), headless inverted (HLI) in the about-facing and forward-facing conditions; dots denote means; ^∗∗∗^*p* < 0.001; ^∗∗^*p* < 0.01; and ^∗^*p* < 0.05.

In the forward-facing condition, for the whole figure images, *d*’ was higher in the WFU condition (*M* = 1.95, *SD* = 0.99) than in the WFI condition (*M* = 1.20, *SD* = 0.92), *t*(27) = 3.19, *p* = 0.004, *d* = 0.60. Interestingly, this was also the case for the headless images as *d*’ was significantly higher in the HLU (*M* = 1.85, *SD* = 0.99) than in the HLI condition (*M* = 1.33, *SD* = 0.85), *t*(27) = 2.07, *p* = 0.048, *d* = 0.39. Therefore, for the forward-facing images, there was a BIE in the whole figure and the headless conditions. The effect size was medium for the whole figure condition and small for the headless condition. Comparing the difference scores between the upright and inverted conditions, revealed that the magnitude of the BIE did not differ significantly between the whole figure (*M* = 0.75, *SD* = 1.24) and the headless conditions (*M* = 0.52, *SD* = 1.32), *t*(27) = 0.73, *p* = 0.471, corrected (α × 2) = 0.942, *d* = 0.14 (see Figure 2).

Further, the magnitude of the inversion effect between the whole figure images in the about-facing and forward-facing conditions was non-significant, *t*(54) = 1.18, *p* = 0.242, corrected (α × 4) = 0.968, *d* = 0.32. For the headless images, the inversion effect between the about-facing and forward-facing conditions was also non-significant with a correction, *t*(54) = 2.10, *p* = 0.040, corrected (α × 4) = 0.160, *d* = 0.57 (see Figure 2). Interestingly, the effect sizes across the about-facing and forward-facing conditions were similar in that for both they were larger in the whole figure than in the headless conditions.

#### Criterion *c* Response Bias

One participant in the about-facing condition had outliers in all four conditions (>2.5 *SD*s) and the scores were replaced with the condition means. Participants were overall more conservative (i.e., greater tendency to report no change or a “same” response in body posture across both same and different trials) in the about-facing than in the forward-facing condition. They were also overall more conservative in the inverted compared to the upright conditions. In the about-facing condition, participants were also significantly more conservative in the HLI than in the HLU condition (see Supplementary Materials for details).

### Efficiency Scores

In the about-facing condition there were three outliers (*z*-score > 2.5 *SD*), one each in the WFI, HLU, and HLI conditions and these were replaced with the condition mean. A 2 × 2 × 2 mixed model ANOVA was performed to compare efficiency scores across the two facing directions (about-facing, forward-facing), between the two body types (whole figure, headless), and the two orientations (upright, inverted). The main effects of facing direction, *F*(1,54) = 0.02, *p* = 0.904, η*_*p*_*^2^ ≤ 0.01, and body type, *F*(1,54) = 1.80, *p* = 0.185, η*_*p*_*^2^ = 0.03, were non-significant. The main effect of orientation was significant, *F*(1,27) = 14.16, *p* ≤ 0.001, η*_*p*_*^2^ = 0.21, as scores were overall more efficient in the upright (*M* = 1234.05, *SD* = 359.04) compared to the inverted conditions (*M* = 1392.49, *SD* = 464.91). There was a non-significant trend for an interaction between body type and orientation, *F*(1,54) = 3.84, *p* = 0.055, η*_*p*_*^2^ = 0.07. The interactions between facing direction and body type, *F*(1,54) = 0.48, *p* = 0.493, η*_*p*_*^2^ = 0.01, facing direction and orientation, *F*(1,54) = 0.65, *p* = 0.425, η*_*p*_*^2^ = 0.01, and facing direction by body type by orientation, *F*(1,27) = 0.05, *p* = 0.825, η*_*p*_*^2^ ≤ 0.01, were non-significant.

**FIGURE 3:**
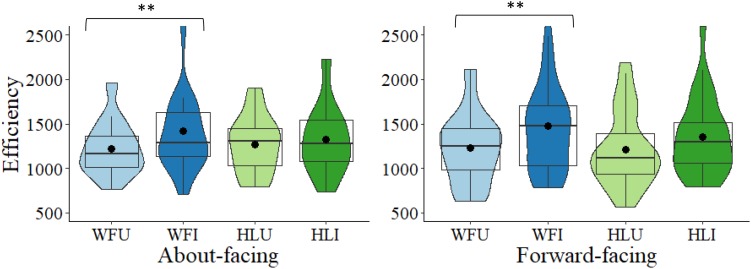
Box and violin plots of efficiency scores for the four conditions: WFU, WFI, HLU, HLI in the about-facing and forward-facing conditions; dots denote means; ^∗∗^*p* < 0.01.

The main question was to determine whether there was a BIE for the different facing directions and body types, despite the non-significant interactions. In the about-facing condition, for the whole figure images, scores were significantly more efficient in the WFU condition (*M* = 1222.35, *SD* = 299.50) than in the WFI condition (*M* = 1416.72, *SD* = 467.04), *t*(27) = −3.32, *p* = 0.003, Cohen’s *d* = −0.63. For the headless images, the difference between HLU (*M* = 1268.59, *SD* = 320.59) and HLI (*M* = 1324.39, *SD* = 380.74) was non-significant, *t*(27) = −0.86, *p* = 0.397, *d* = −0.16. Therefore, for the about-facing images, there was a BIE in the whole figure, but not the headless conditions, which is also reflected in the effect sizes, with a medium effect size in the whole figure condition and small in the headless condition. Differences scores were then calculated between the upright and inverted conditions. A comparison of the difference scores between the whole figure and headless conditions, revealed that the magnitude of the difference between the upright and inverted images (i.e., the BIE) was non-significant between the whole figure (*M* = 193.37, *SD* = 308.40) and the headless conditions (*M* = 55.80, *SD* = 343.40), when a correction was applied, *t*(27) = 2.15, *p* = 0.041, corrected (α × 2) = 0.082, *d* = 0.41 (see Figure 3).

In the forward-facing condition, participants were significantly more efficient in the WFU condition (*M* = 1230.34, *SD* = 394.53) than in the WFI condition (*M* = 1477.41, *SD* = 546.96), *t*(27) = −3.07, *p* = 0.005, *d* = −0.58; whereas for the headless images, the difference between HLU (*M* = 1214.93, *SD* = 421.56) and HLI (*M* = 1352.46, *SD* = 431.15) was non-significant, *t*(27) = −1.53, *p* = 0.137, *d* = −0.29. Therefore, for the forward-facing images, there was a BIE in the whole figure, but not the headless conditions. Similarly, the effect was medium in the whole figure condition and small in the headless condition. Based on the difference scores between the upright and inverted conditions, the magnitude of the difference between the upright and inverted images (i.e., the BIE) did not differ significantly between the whole figure (*M* = 247.07, *SD* = 425.91) headless conditions (*M* = 137.53, *SD* = 474.69), *t*(27) = 1.01, *p* = 0.322, corrected (α × 2) = 0.644, *d* = 0.19 (see Figure 3).

Further, the difference in the magnitude of the inversion effect between the whole figure images in the about-facing and forward-facing conditions was non-significant, *t*(54) = 0.54, *p* = 0.591, corrected (α × 4) = 1.00, *d* = 0.14. Likewise, for the headless images, the difference in the magnitude of the inversion effect between the about- and forward-facing conditions was non-significant, *t*(54) = 0.74, *p* = 0.464, corrected (α × 4) = 0.928, *d* = 0.20 (see Figure 3).

### Dwell Time to Heads, Bodies, and Feet

Polygonal interest areas (IAs) were created around the head, body, and feet of each image using Data Viewer software version 3.2.48 (SR Research). IAs were created around the feet because in the inverted images, the feet appear in the region where the head would normally appear. The IAs around the heads included the head and neck, the IAs around the bodies extended from the top of the torso to the ankles, and the feet IAs were around the feet of the figures. Given that there is a bias to look in the upper region (e.g., Arizpe et al., 2017), it was expected that participants might look to that region in the inverted condition. Dwell time (DT) refers to the summed durations of all the fixations within an IA. DT to the IAs of the test image was averaged across all trials in each condition. Proportional DTs to each IA (head, body, feet) were calculated by dividing looking to each IA by looking to all three IAs [e.g., DT to head/(head + body + feet)]. Proportional DT to the heads, bodies, and feet were compared separately across conditions to avoid violating the assumption of independence given that heads, bodies and feet appear simultaneously. Following this, for each figure type, the relationships between DT proportions to each IA and efficiency scores were analyzed to assess whether the proportion of time spent looking at particular areas was related to performance. Efficiency scores were used instead of *d*’ because *d*’ had restricted range making it less suitable for correlations.

#### Heads (Head and Neck)

**FIGURE 4:**
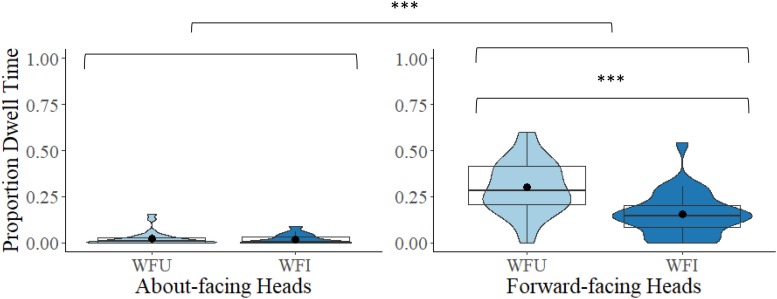
Box and violin plots of proportional dwell time (DT) to the heads (head and neck of the figures) in the WFU and WFI conditions in the about-facing and forward-facing conditions; dots denote means; ^∗∗∗^*p* < 0.001.

As there was largely no looking at the head region of the headless stimuli, only the whole figure conditions were included in this analysis. A 2 × 2 mixed model ANOVA comparing proportional DT to the heads between the two facing directions (about-facing, forward-facing) and between the two orientations (WFU, WFI) revealed a main effect of facing direction, *F*(1,54) = 84.90, *p* < 0.001, η*_*p*_*^2^ = 0.61. Proportional DT to the heads was larger in the forward-facing (*M* = 0.23, *SD* = 0.13) than in the about-facing experiment (*M* = 0.02, *SD* = 0.03). The main effect of orientation was also significant, *F*(1,54) = 35.69, *p* < 0.001, η*_*p*_*^2^ = 0.40. There was overall greater looking at the heads in the upright (*M* = 0.16, *SD* = 0.09) compared to inverted whole figures (*M* = 0.09, *SD* = 0.07). There was also a significant interaction between facing direction and orientation, *F*(1,54) = 32.75, *p* < 0.001, η*_*p*_*^2^ = 0.38. For the about-facing experiment, the difference in proportional DT to the heads of the upright and inverted whole figures was non-significant, *t*(27) = 0.34, *p* = 0.734, *d* = 0.07, but for the forward-facing condition, proportional DT was significantly larger to the heads of the upright than the inverted whole figures, *t*(27) = 6.29, *p* < 0.001, *d* = 1.19 (see Figure 4).

#### Bodies (Top of Torso to Ankles)

**FIGURE 5:**
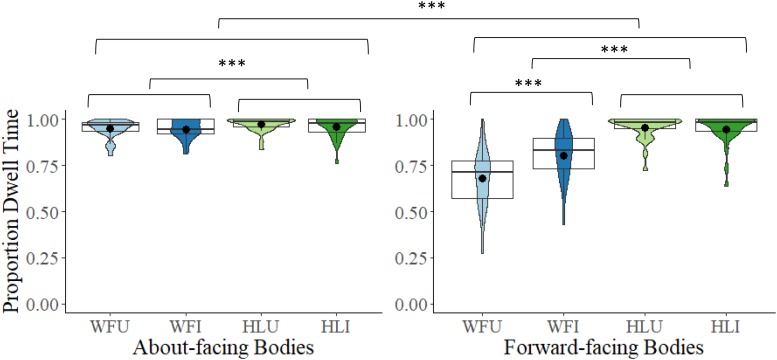
Box and violin plots of proportional dwell time (DT) to the bodies (top of torso to ankles) in the WFU, WFI, HLU, and the HLI conditions in the about-facing and forward-facing conditions; dots denote means; ^∗∗∗^*p* < 0.001.

A 2 × 2 × 2 mixed model ANOVA comparing proportional DT to the bodies between the two facing directions (about-facing, forward-facing), the two body types (whole figure, headless), and the two orientations (upright, inverted) revealed a main effect of facing direction, *F*(1,54) = 34.21, *p* < 0.001, η*_*p*_*^2^ = 0.39. Proportional DT to the bodies was larger in the about-facing (*M* = 0.96, *SD* = 0.05) than in the forward-facing condition (*M* = 0.85, *SD* = 0.11). There was also a main effect of body type, *F*(1,54) = 121.32, *p* < 0.001, η*_*p*_*^2^ = 0.69. Proportional DT to the bodies was larger in the headless (*M* = 0.96, *SD* = 0.06) than in the whole figure condition (*M* = 0.84, *SD* = 0.10). There was also a main effect of orientation, *F*(1,54) = 12.13, *p* < 0.001, η*_*p*_*^2^ = 0.18. Proportional DT to the bodies was larger in the inverted (*M* = 0.91, *SD* = 0.08) than in the upright condition (*M* = 0.89, *SD* = 0.08). The interactions between facing direction and body type, *F*(1,54) = 87.34, *p* < 0.001, η*_*p*_*^2^ = 0.62, facing direction and orientation, *F*(1,54) = 23.45, *p* < 0.001, η*_*p*_*^2^ = 0.30, and body type and orientation, *F*(1,54) = 27.77, *p* < 0.001, η*_*p*_*^2^ = 0.34, were all significant, as was the interaction between facing direction, body type, and orientation, *F*(1,54) = 20.50, *p* < 0.001, η*_*p*_*^2^ = 0.28. These interactions were explored further. There was significantly greater looking at whole figure bodies (upright and inverted) in the about-facing than in the forward-facing conditions (*p*s < 0.001, corrected (α × 4) ≤ 0.001, *d*s > 1.43, see Figure 5), but there was no difference found for headless conditions (upright and inverted, *p*s > 0.567, corrected (α × 4) = 1.00). For the about-facing experiment, Bonferroni-corrected *post hoc* comparisons revealed that there was significantly greater looking at the bodies in the HLU condition than in the WFU (*p* = 0.003, corrected (α × 6) = 0.019, *d* = 0.61) and WFI conditions (*p* = 0.005, corrected (α × 6) = 0.027, *d* = 0.58). The difference in looking at bodies between the upright and inverted headless conditions was non-significant (*p* = 1.00). However, there was significantly greater looking at bodies in the WFI than in the WFU condition (*p* < 0.001, corrected (α × 6) < 0.001, *d* = 1.05. For the forward-facing experiment, there was significantly greater looking at the bodies in the headless (upright and inverted) conditions than in the whole figure (upright and inverted) conditions (*p*s < 0.001, corrected (α × 6) < 0.001, *d*s > 1.29). The difference in looking at bodies between the upright and inverted headless conditions was non-significant (*p* = 1.00). However, there was significantly greater looking at bodies in the WFI than in the WFU condition (*p* < 0.001, corrected (α × 6) < 0.001, *d* = 1.05, see Figure 5).

#### Feet (From Ankles and Bottom of Feet)

**FIGURE 6:**
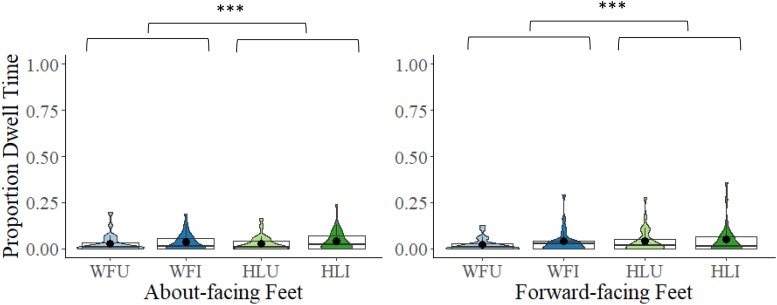
Box and violin plots of proportional dwell time (DT) to the feet (from ankles to bottom of feet) in the WFU, WFI, HLU, and the HLI conditions in the about-facing and forward-facing conditions; dots denote means; ^∗∗∗^*p* < 0.001.

A 2 × 2 × 2 mixed model ANOVA comparing proportional DT to the feet between the two facing directions (about-facing, forward-facing), the two body types (whole figure, headless), and the two orientations (upright, inverted) revealed that the main effect of facing direction was non-significant, *F*(1,54) = 0.31, *p* = 0.581, η*_*p*_*^2^ = 0.01. The main effect of body type was significant, *F*(1,54) = 5.10, *p* = 0.028, η*_*p*_*^2^ = 0.09. Proportional DTs were overall greater to the feet in the headless (*M* = 0.04, *SD* = 0.06) than in the whole figure conditions (*M* = 0.03, *SD* = 0.05). The main effect of orientation was non-significant, *F*(1,54) = 2.83, *p* = 0.098, η*_*p*_*^2^ = 0.05. The interactions between facing direction and body type, *F*(1,54) = 2.47, *p* = 0.122, η*_*p*_*^2^ = 0.04, facing direction and orientation, *F*(1,54) = 0.02, *p* = 0.880, η*_*p*_*^2^ = 0.01, and body type and orientation, *F*(1,54) = 0.12, *p* = 0.733, η*_*p*_*^2^ = 0.01, were all non-significant, as was the interaction between facing direction, body type, and orientation, *F*(1,54) = 1.97, *p* = 0.167, η*_*p*_*^2^ = 0.04 (see Figure 6).

#### Relationship Between DT Proportions and Performance

For each condition, the relationship between the DT proportions to each IA and efficiency scores was analyzed to see whether the proportion of time spent looking at particular areas was associated with performance (see Table 1). Pearson’s *r* correlations were performed and for the whole figures Bonferroni corrections were applied based on the presence of three IAs (α × 3) and for the headless images, Bonferroni corrections were based on the presence of two IAs (α × 2).

**TABLE 1:** Pearson’s *r* correlations between efficiency scores and proportional dwell time to heads, bodies, and feet.

**About-facing efficiency**	**Dwell time (DT) proportion**			**Forward-facing efficiency**	**Dwell time (DT) proportion**		
	**Heads**	**Bodies**	**Feet**		**Heads**	**Bodies**	**Feet**
Whole figure upright				Whole figure upright			
Pearson’s *r*	−0.002	0.118	−0.132	Pearson’s *r*	0.226	−0.257	0.283
(*p*-value)	(0.992)	(0.548)	(0.503)	(*p*-value)	(0.257)	(0.196)	(0.153)
Corrected *p*^∗^	(0.999)	(0.999)	(0.999)	Corrected *p*^∗^	(0.771)	(0.588)	(0.459)
95% CIs	−0.375–0.371	−0.266–0.471	−0.481–0.254	95% CIs	−0.168–0.558	−0.580–0.137	−0.109–0.598
Whole figure inverted				Whole figure inverted			
Pearson’s *r*	0.436	−0.539	0.329	Pearson’s *r*	0.484	−0.499	0.080
(*p*-value)	(0.020)	(0.003)	(0.088)	(*p*-value)	(0.011)	(0.008)	(0.693)
Corrected *p*^∗^	(0.060)	(0.009)	(0.264)	Corrected *p*^∗^	(0.033)	(0.024)	(0.999)
95% CIs	0.075–0.696	−0.759 to−0.207	−0.051–0.625	95% CIs	0.127–0.730	−0.958 to−0.807	−0.310–0.446
Headless upright				Headless upright			
Pearson’s *r*	–	−0.102	0.075	Pearson’s *r*	–	−0.215	0.213
(*p*-value)		(0.607)	(0.703)	(*p*-value)		(0.282)	(0.287)
Corrected *p*^∗^		(0.999)	(0.999)	Corrected *p*^∗^		(0.564)	(0.574)
95% CIs		−0.457–0.282	−0.309–0.436	95% CIs		−0.550–0.180	−0.182–0.548
Headless inverted				Headless inverted			
Pearson’s *r*	–	−0.208	0.209	Pearson’s *r*	–	−0.423	0.432
(*p*-value)		(0.288)	(0.286)	(*p*-value)		(0.028)	(0.024)
Corrected *p*^∗^		(0.576)	(0.572)	Corrected *p*^∗^		(0.056)	(0.048)
95% CIs		−0.539–0.179	−0.178–0.540	95% CIs		−0.692 to −0.051	0.062–0.697

*^∗^Bonferroni corrections (α × 3 for each whole figure condition and α × 2 for each headless condition).*

#### Whole Figure Upright Images

In both the about- and forward-facing conditions, there were no significant relationships between DT proportions to any of the IAs and efficiency scores, *r*s < 0.257, *p*s > 0.196.

#### Whole Figure Inverted Images

In both the about- and forward-facing conditions, there was a significant negative relationship between DT proportions to the bodies and efficiency scores, *r*s > −0.499 (Bonferroni-corrected *p*s < 0.008). In the forward-facing condition there was a significant positive relationship between DT to the heads, *r* = 0.484 (Bonferroni-corrected *p* = 0.033); and in the about-facing condition there was a similar non-significant trend *r* = 0.436 (Bonferroni-corrected *p* = 0.060). These findings suggest that when the whole figures are inverted, the more participants look at the bodies and the less they look at the heads, the better their performance.

#### Headless Upright Images

In both the about- and forward-facing conditions, there were no significant relationships between DT proportions to any of the IAs and efficiency scores (see Table 1).

#### Headless Inverted Images

In the about-facing condition, there were no significant relationships between DT proportions and efficiency scores. In the forward-facing condition, there was a significant positive relationship between looking at the feet and efficiency scores, *r* = 0.432 (Bonferroni-corrected *p* = 0.048) and a non-significant negative trend for looking the bodies and efficiency scores *r* = −0.423 (Bonferroni-corrected *p* = 0.056). This suggests that the less participants looked at the feet and the more they looked at the bodies the better the performance.

## Discussion

Analyzing both *d*’ and efficiency scores revealed BIEs with the whole figures in both the about- and forward-facing images, replicating previous studies (Minnebusch et al., 2009; Brandman and Yovel, 2010, 2012; Yovel et al., 2010; Robbins and Coltheart, 2012b; Arizpe et al., 2017). For the headless images, there was no BIE for forward-facing images with efficiency scores, but there was a BIE with *d*’ scores, again like previous studies which have sometimes showed BIEs for headless bodies and sometimes not (e.g., Yovel et al., 2010; Arizpe et al., 2017). The never before tested about-facing headless bodies showed no BIE with either measure.

Therefore, we do find a forward-facing headless BIE with *d*’ scores, but the magnitude of the BIE did not differ significantly between the whole figure and headless conditions. For the about-facing condition, the magnitude of the BIE (based on *d*’ scores) was larger in the whole figure compared to the headless conditions, due to the absence of a BIE in the about-facing headless condition. Participants were also more conservative, responding “same,” whether correct or incorrect, in the about-facing inverted headless than in the upright headless condition. As there was a forward-facing headless BIE, this suggests that even without head information, when people are seen from a frontal view, the advantage of seeing the images in an upright compared to an inverted orientation leads to similar effects as is seen with whole figures. Note, effect sizes in both the about-facing and forward-facing conditions tended to be medium for the whole figures and small for the headless figures. This is consistent with the findings of Arizpe et al. (2017).

Brandman and Yovel (2012) suggested that the BIE might be based on the presence or induced presence of a face and provided evidence that people were more likely to imagine faces in briefly presented stimuli with images that were also found to have larger BIEs, namely (faceless) forward-facing whole figures, suggesting a role of contextual priming. The condition that did not fit with that trend in their study was the about-facing whole figures, for which people did not imagine a face, but which showed a BIE, albeit smaller in magnitude than in the forward-conditions. An extension to this interpretation might be that BIEs are weaker for bodies presented in less typical forms (i.e., about-facing, headless), which would predict a BIE for whole figures seen from behind, but smaller BIE for bodies without heads. Unlike Brandman and Yovel (2010) we failed to see any difference in BIEs between the forward- and about-facing conditions and we also find a headless BIE in the forward-facing condition (with *d*’ only). Performance was overall better in the forward-facing conditions suggesting that faces (present or induced in the case of headless bodies) might contribute to better body posture discrimination. We also see a larger BIE in the about-facing whole figure compared to the headless condition. This pattern of BIEs, suggests that the BIE weakens as bodies appear in a less typically experienced form and in forms where heads are least likely to be induced, such as in headless, about-facing presentations.

The question of contextual priming raises another related question, that of repetition priming, or a combination of repetition and contextual priming. If bodies are seen with a head and then without a head there is a chance that the face is more likely to be perceived. In the current study, and most others, this is the design. The whole and headless figures were presented within-groups so that the relative size of the BIEs could more accurately be compared. Yovel et al. (2010) and Brandman and Yovel (2010, 2012) used between groups designs to reduce the chance of such priming, with the major downside being that the groups were small. We also tested one of our conditions between groups – that of forward- versus about-facing. If priming were the only thing leading to a BIE for headless figures, then it seems likely that we should have found some indication of this for about-facing as well as forward-facing figures, but we did not as there was no BIE in the about-facing headless condition. This further supports our argument that the BIE is strongest for the most prototypical or most frequently experienced body – forward-facing and with a head. Reed et al. (2006) showed that BIEs were larger for whole intact bodies than scrambled bodies. Reed et al. (2012) tested the role of experience in a different way showing people computer-generated human or dog figures in human or dog poses. The most commonly experienced canonical poses (humans in human poses) showed the largest inversion effects, whereas inversion effects were smaller for dogs in human poses and humans in dog poses. Interestingly, there were no inversion effects for dogs in dog poses, which Reed at al. interpret as showing that it is the embodied experience as well as the visual experience that matters.

What role then does facial or even induced facial information play? As mentioned, Brandman and Yovel (2012) found that participants reported seeing (absent) facial features at higher rates in forward-facing than in about-facing whole figures for very briefly presented stimuli. Here using photographic images of people, proportional looking to the heads was larger in the forward- than the about-facing images and larger for the upright than the inverted forward-facing heads. This was despite participants engaging in *body* posture discrimination task and the head information between the pairs being identical. Looking at heads should have conferred no advantages. There was also less looking at bodies in the forward-facing upright images than inverted images or about-facing images, and overall less looking at bodies in the upright compared to the inverted images. Nonetheless, there were better *d*’ scores in the forward- than in the about-facing condition and better scores in the upright compared to the inverted whole figures. Therefore, greater looking times at the bodies does not necessarily explain participants’ performance. Does this mean that greater looking to the heads instead explains participants’ performance? Correlations revealed that there was no direct evidence for greater DT proportions to heads and better performance.

There was also a headless BIE in the forward-, but not the about-facing conditions (for *d*’ scores). Heads are of course absent in the headless condition, but heads are attached to bodies. Therefore, faces might be more easily induced with forward-facing than with about-facing headless bodies. As mentioned, directly looking at heads might not be necessary for a BIE given that the correlations between DT proportions to heads and performance were weak and we find a headless BIE (forward-facing) and a BIE with about-facing whole figures. Arizpe et al. (2017), found that focusing on the upper torso or head in both orientations was associated with better performance, so it might be a combination of the two areas. We also found that in the HLI condition (forward-facing), the longer the DT proportion to the feet the poorer the performance, which is similar to Arizpe et al. (2017). Brandman and Yovel (2010) did not ask participants if they saw faces in briefly presented inverted images, so it is uncertain how much faces are perceived in inverted images, but presumably less so than with upright images; and this might explain the poorer performance with inverted figures.

What we did find is that with inverted whole figures, a greater focus on bodies and less on heads was associated with better performance, particularly in the forward-facing condition. Our findings, therefore, also suggest that when the task is more difficult as it is with a less typical body format such as inverted bodies, a greater focus on bodies and less on heads or feet is associated with better performance. Therefore, when upright, the more easily induced the face and the more typical the images, the better the performance; and when inverted a stronger focus on the bodies is associated with better performance.

Inversion effects are often taken as an indirect measure of holistic or configural processing (see extensive discussion of this in Robbins and McKone, 2007). Although inversion effects are found for many stimuli with a canonical upright orientation, they tend to be stronger for faces and human bodies (e.g., Reed et al., 2003; Yovel et al., 2010; Robbins and Coltheart, 2012b). A few studies have also used a more direct measure of holistic processing in bodies, the composite task. In the original version of this task, top and bottom halves from two different faces are combined and it is harder to name one half when the two are aligned than when they are misaligned (Young et al., 1987). In the matching version of this task, participants are asked to say whether the top, bottom, left or right halves of a pair of items are the same or different while ignoring the other half, and again the task is harder for aligned than misaligned stimuli (e.g., Robbins and McKone, 2007). Robbins and Coltheart (2012a) found that whole bodies with heads, but obscured faces, are holistically processed in the matching version of the composite task. Similarly, Willems et al. (2014) found composite effects for bodies with heads (showing faces) in a posture matching task. Bauser et al. (2011) found no holistic processing for bodies with heads or without heads on the composite task, but the clothes were so different that the task could be done without looking at identity, making the lack of a composite effect less surprising. Thus, it is not known whether there is a composite effect for headless bodies. As for findings that a BIE is typically found with figures with more easily induced faces, results from the composite task could be because faces are induced, which contributes to holistic/configural processing of bodies when upright due to activation of the face-selective brain regions as first proposed by Brandman and Yovel (2010) and Yovel et al. (2010).

One limitation of this study is that we did not have faceless, forward-facing whole figures making it difficult to compare our findings to others, which largely had faceless heads (e.g., Yovel et al., 2010; Arizpe et al., 2017). Participants here looked longer at the forward- than at the about-facing heads. By including a faceless forward-facing condition, we could also more directly compare the effect of the presence of faces on the BIE in forward-facing images. Further, Brandman and Yovel (2012) found a significantly smaller BIE in the about- compared to the forward-facing images while we did not find a difference. Both this study and theirs was between groups for facing direction and perhaps a within groups design could help to elucidate this difference.

## Conclusion

In conclusion, consistent with the literature (e.g., Brandman and Yovel, 2012; Arizpe et al., 2017), we find a consistent BIE with whole figures whether they are about- or forward-facing. The BIE with the headless images was less consistent. Arizpe et al. (2017) argued that the headless BIE is weaker than the whole figure BIE and the presence or absence of a headless BIE is more susceptible to statistical power. The sample size here was likely sufficient as the forward-facing headless BIE was similar in magnitude to the forward-facing whole figure BIE, but there were weaker effect sizes in the headless conditions. In the about-facing condition, the magnitude of the BIE was smaller (and absent) for the headless than for the whole figure images. Therefore, the presence or strength of the BIE is likely due to a combination of the likelihood that a face can be induced and the more prototypical the format of the figure is (whole figure, upright, or forward-facing for headless images). Faces are more easily induced with more prototypical presentations such as with WFU images (Brandman and Yovel, 2012). A further unique finding here is that participants looked at the forward-facing heads more than the about-facing heads even though the heads of the test pairs in each condition were identical and therefore were not informative for the task. However, looking at heads was not directly associated with better performance. Participants also looked more at the inverted than the upright bodies and the headless than the whole figure bodies. Greater looking at inverted whole figure bodies was associated with better performance, but the relationship between looking at upright bodies and performance was weak. Therefore, a focus on faces or bodies *per se* does not explain performance. Instead, a BIE is more reliable when the figures are seen in their most typical format, and when the face is more easily induced, as is the case with upright images, This might lead to better performance as configural processing might be stronger, which in turn might lead to better discrimination. When the task is more difficult, as with inverted images, a focus on bodies is associated with better performance.

## Data Availability Statement

The datasets generated for this study are available on request to the corresponding author.

## Ethics Statement

The studies involving human participants were reviewed and approved by the ANU Human Research Ethics Committee. The patients/participants provided their written informed consent to participate in this study.

## Author Contributions

EA, RR, and HFC wrote the manuscript. EA and HFC were involved in the study design and analyses. HFC and HWC collected the data. EA, RR, HFC, and HWC contributed intellectually to the manuscript.

## Conflict of Interest

The authors declare that the research was conducted in the absence of any commercial or financial relationships that could be construed as a potential conflict of interest.

## Footnotes

1 www.shutterstock.com

2 All subsequent corrections are Bonferroni-corrected.
