# Supplementary material for: Body Inversion Effects With Photographic Images of Body Postures: Is It About Faces?
Source: Front Psychol. 2019 Nov 29;10:2686. doi: 10.3389/fpsyg.2019.02686 (PMC6896224; doi:10.3389/fpsyg.2019.02686)
Supplement: Supplementary file 1 [file Data_Sheet_1.PDF]

Supplementary Material

Criterion *c* Response Bias.

A 2 × 2 × 2 mixed model ANOVA comparing *c* criterion scores across the two facing directions (about-facing, forward-facing), the two body types (whole figure, headless), and the two orientations (upright, inverted) revealed that there was a main effect of facing direction, $F(1,54) = 6.41$, $p = .014$, $\eta_p^2 = .16$. Participants were overall more conservative (i.e., greater tendency report no change or a 'same response' in body posture across both same and different trials) in the about-facing ($M = -0.32$, $SD = 0.36$) than in the forward-facing condition ($M = -0.54$, $SD = 0.50$, see Supplementary Figure 1). The main effect of body type was non-significant, $F(1,54) = 2.45$, $p = .123$, $\eta_p^2 = .04$, but there was a significant main effect of orientation, $F(1,54) = 4.08$, $p = .048$, $\eta_p^2 = .07$. Criterion *c* scores were overall more conservative in the inverted ($M = -0.38$, $SD = 0.43$) compared to the upright condition ($M = -0.48$, $SD = 0.42$). The interactions between facing direction and body type, $F(1,54) = 0.85$, $p = .360$, $\eta_p^2 = .02$, facing direction and orientation, $F(1,54) = 0.07$, $p = .799$, $\eta_p^2 = .01$, and body type and orientation, $F(1,54) = 0.77$, $p = .384$, $\eta_p^2 = .01$, were all non-significant. The facing direction by body type by orientation interaction was non-significant, $F(1,54) = 0.76$, $p = .387$, $\eta_p^2 = .01$.

In the about-facing condition, the difference between the whole figure upright ($M = -0.31$, $SD = 0.31$) and the whole figure inverted conditions ($M = -0.30$, $SD = 0.34$), was non-significant, $t(27) = -0.15$, $p = .884$, $d = -0.03$. However, participants were significantly more conservative in the headless inverted ($M = -0.25$, $SD = 0.37$) than in the headless upright condition ($M = -0.42$, $SD = 0.41$), $t(27) = 2.38$, $p = .025$, $d = 0.45$. The magnitude of the difference between the upright and inverted images (i.e., the BIE) in the whole figure ($M = -0.01$, $SD = 0.35$) and the headless conditions ($M = -0.17$, $SD = 0.38$), was non-significant,

t(27) = -1.86, *p* = .074, Bonferroni-corrected[1] (α × 2) = .148, *d* = -0.35 (see Supplementary Figure 1).

In the forward-facing condition, for the whole figure images, the difference between the whole figure upright (*M* = -0.54, *SD* = 0.48) and the whole figure inverted conditions (*M* = -0.43, *SD* = 0.57), was non-significant, *t*(27) = -0.94, *p* = .367, *d* = -0.18. The difference between the headless upright (*M* = -0.65, *SD* = 0.50) and the headless inverted conditions (*M* = -0.53, *SD* = 0.46), was also non-significant, *t*(27) = -1.02, *p* = .317, *d* = -0.19. The magnitude of the difference between the upright and inverted images did not differ significantly between the whole figure (*M* = -0.12, *SD* = 0.66) and the headless conditions (*M* = -0.12, *SD* = 0.61), *t*(27) = -0.01, *p* = .998, corrected (α × 2) = 1.00, *d* = 0.00 (see Supplementary Figure 1).

Further, the magnitude of the inversion effect between the whole figure images in the about-facing and forward-facing conditions was non-significant, *t*(54) = 0.76, *p* = .450, corrected (α × 4) = 1.00, *d* = 0.20. For the headless images, the inversion effect between the about-facing and forward-facing conditions was also non-significant, *t*(54) = -0.40, *p* = .690, corrected (α × 4) = 1.00, *d* = -0.11.

[1] All subsequent corrections are Bonferroni-corrected.

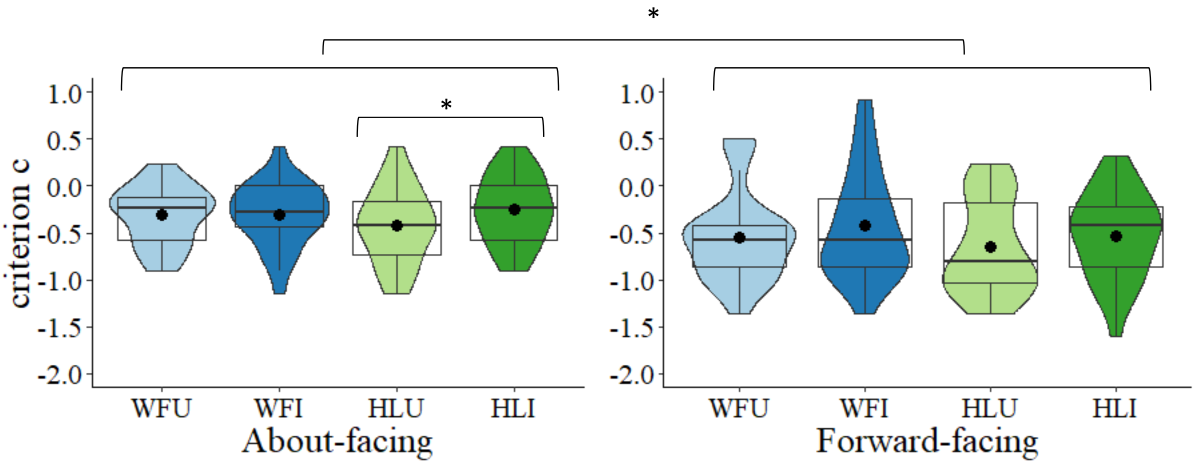

Supplementary Figure 1. Box and violin plots of criterion *c* scores for the four conditions: whole figure upright (WFU), whole figure inverted (WFI), headless upright (HLU), headless inverted (HLI) in the about-facing and forward-facing conditions; dots denote means; *** = p <.001; ** = $p < .01$; * = $p < .05$
